# Characterisation of the cellular and proteomic response of *Galleria mellonella* larvae to the development of invasive aspergillosis

**DOI:** 10.1186/s12866-018-1208-6

**Published:** 2018-06-28

**Authors:** Gerard Sheehan, Gráinne Clarke, Kevin Kavanagh

**Affiliations:** Department of Biology, Maynooth University, Maynooth, Co. Kildare Ireland

**Keywords:** *Aspergillus*, *Galleria*, Infection, Immunity, Invasive aspergillosis, Granuloma, Mini-model

## Abstract

**Background:**

*Galleria mellonella* larvae were infected with conidia of *Aspergillus fumigatus* and the cellular and humoral immune responses of larvae to the pathogen were characterized as invasive aspergillosis developed.

**Results:**

At 2 h post-infection there was an increase in hemocyte density to 7.43 ± 0.50 × 10^6^/ml from 0.98 ± 0.08 × 10^6^/ml at 0 h. Hemocytes from larvae immune primed for 6 h with heat killed *A. fumigatus* conidia displayed superior anti-fungal activity. Examination of the spread of the fungus by Cryo-imaging and fluorescent microscopy revealed dissemination of the fungus through the larvae by 6 h and the formation of distinct nodules in tissue. By 24 h a range of nodules were visible at the site of infection and at sites distant from that indicating invasion of tissue. Proteomic analysis of larvae infected with viable conidia for 6 h demonstrated an increase in the abundance of gustatory receptor candidate 25 (37 fold), gloverin-like protein (14 fold), cecropin-A (11 fold). At 24 h post-infection gustatory receptor candidate 25 (126 fold), moricin-like peptide D (33 fold) and muscle protein 20-like protein (12 fold) were increased in abundance. Proteins decreased in abundance included fibrohexamerin (13 fold) and dimeric dihydrodiol dehydrogenase (8 fold).

**Conclusion:**

The results presented here indicate that *G. mellonella* larvae may be a convenient model for studying the stages in the development of invasive aspergillosis and may offer an insight into this process in mammals.

**Electronic supplementary material:**

The online version of this article (10.1186/s12866-018-1208-6) contains supplementary material, which is available to authorized users.

## Background

*Galleria mellonella* larvae are an ideal in vivo model to quickly and easily assess the virulence of a range of human pathogens, to comprehensively analyze the host – pathogen interactome and to evaluate the in vivo toxicity and efficacy of antimicrobial agents [1–3]. *G. mellonella* larvae are easy to inoculate, generate results within 48 h and are free from the ethical and legal restrictions which surround the use of mammals for this type of testing [4, 5]. Microbial virulence and pathogenesis can be assessed using a variety of endpoints including survival, fluctuations in hemocyte density, oscillations in fungal burden and changes in hemolymph proteome [6, 7]. Insect hemocytes can be easily isolated and used in ex vivo cellular assays to determine phagocyte – pathogen interactions [8].

*G. mellonella* larvae have been used to assess the virulence of a range of fungal pathogens including *Aspergillus fumigatus, Candida albicans* and *Cryptococcus neoformans* and generated results with strong correlations to those established in mammals [9–11]. The generation of comparable results is due to many similarities which exist between the mammalian innate immune response and the insect immune system [5, 12]. Insect hemocytes have receptors (Toll, β-1,3-glucan, etc) and signaling pathways (IMD, JNK, JAK/STAT) similar to those of mammalian neutrophils [12, 13]. Hemocytes can engage in lectin mediated phagocytosis of opsonized microbial cells. Hemocytes can also incorporate proteins homologous to neutrophil p40^phox^, p47^phox^, p67^phox^ and gp91^phox^ into their membranes which contribute to the formation of an active NADPH oxidase complex capable of producing superoxide [8, 14]. Hemocytes also undergo degranulation and release a range of granular proteins into the phagosome [12]. Extracellular killing is mediated by degranulation, the formation of NET-like structures and the formation of macro-cellular structures (melanotic encapsulation and nodulation) homologous to mammalian complement proteins and granuloma formation [8, 15–18]. Furthermore, *A. fumigatus* toxins gliotoxin and fumagillin inhibit the microbicidal activity of human neutrophils and insect hemocytes by blocking the formation of F-actin [19, 20].

*A. fumigatus* can induce allergic, saprophytic and invasive disease depending upon the host immune status. In the case of invasive disease inhaled conidia germinate, form hyphae within alveoli and migrate beyond the pulmonary epithelium and into the bloodstream (angioinvasive), ultimately disseminating throughout the host if anti-fungal therapy is not commenced [21, 22]. In the murine model of chronic granulomatous disease (CGD), invading hyphae are found within granulomatous lesions predominated by neutrophils that probably function to prevent spread of conidia [23]. Larvae of *G. mellonella* have been utilized to study the virulence of *A. fumigatus* and provided results comparable to those from murine studies. *A. fumigatus Δpes*3 mutants displayed significantly higher mortality in *G. mellonella* than WT *A. fumigatus* which was mirrored in two murine models of pulmonary aspergillosis (corticosteroid-treated and neutropenic) [24]. Mutant strains of *A. fumigatus* (Δ*sid*A, Δ*sid*C, Δ*sid*D, Δ*sid*F, Δ*paba* and Δ*cpcA*) demonstrated almost complete correlation of virulence when assessed in *G. mellonella* and mice [9]. Hemocytes can discriminate between non-germinated, germinating and hyphal forms of *A. fumigatus* which is also the case with human neutrophils [25, 26]. *Aspergillus terreus* infection in *G. mellonella* demonstrated unique histological findings consistent with those observed in disseminated aspergillosis in mammals [27].

The aim of the work presented here was to analyze the response of *G. mellonella* larvae to *A. fumigatus* infection and to examine similarities with invasive aspergillosis in mammals. While *G. mellonella* larvae are now widely used as in vivo models a greater understanding of the pathogen – host interaction may allow the identification of a range of end points and further validate use of larvae for studying disease processes in vivo.

## Results

### Responses of *G. mellonella* larvae to *A. fumigatus* infection

**Fig. 1:**
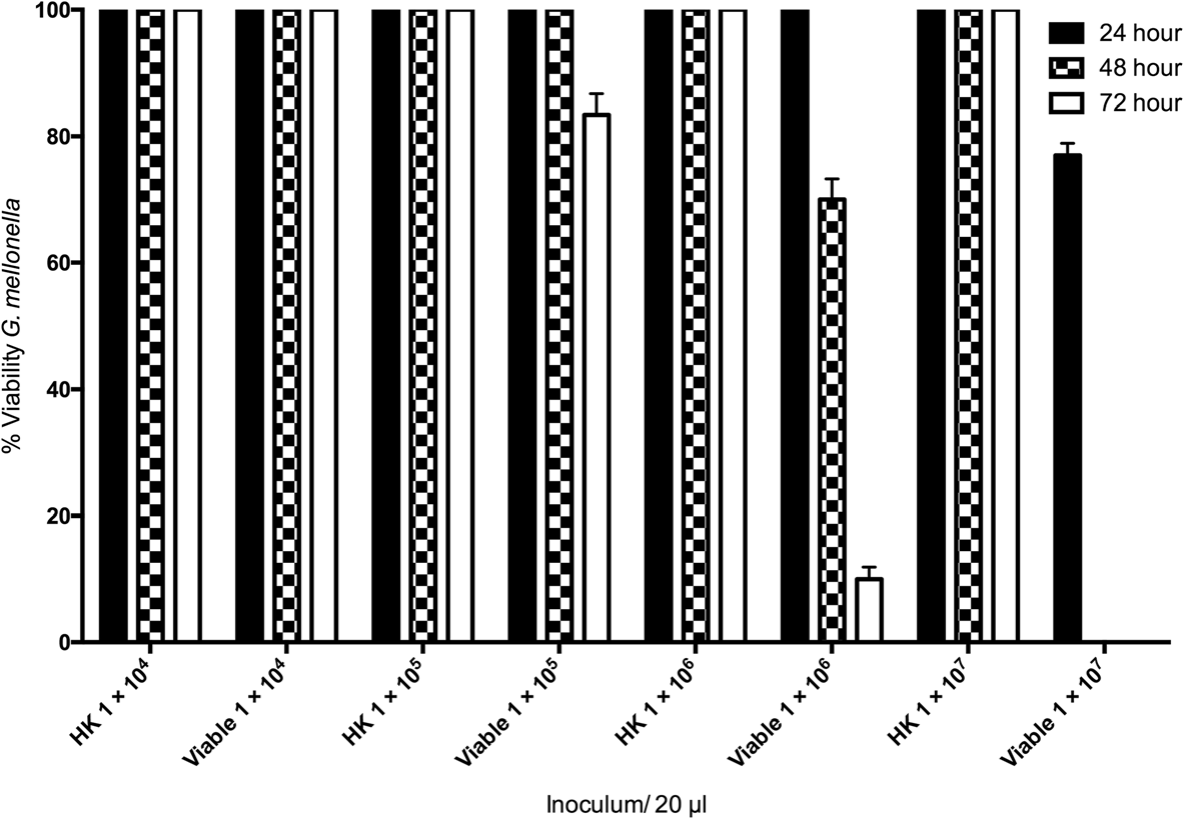
Effect of viable and heat killed (HK) *A. fumigatus* conidia on viability of *G. mellonella* larvae over 72 h. *G. mellonella* larvae were inoculated with 20 μl *A. fumigatus* at doses ranging from 1 × 10^4^ to 1 × 10^7^ incubated at 37 °C and viability assessed over 72 h. All values are the mean ± S.E of three independent experiments

Larvae were inoculated with heat killed (HK) and viable conidia of *A. fumigatus*, incubated at 37 °C and viability assessed over 72 h. An inoculum of heat killed conidia ranging from 1 × 10^4^ to 1 × 10^7^/ larva yielded no change in larval viability over 72 h. An inoculum of 1 × 10^4^ viable conidia/ larva resulted in no change in larval viability over 72 h whereas 1 × 10^5^ viable conidia/larva caused 16.7 ± 5.7% mortality after 72 h. A dose of 1 × 10^6^ viable conidia resulted in 30 ± 5.7% and 90 ± 3.3% mortality after 48 h and 72 h respectively. Inoculation of larvae with conidia at a dose of 1 × 10^7^/ larva reduced viability to 77 ± 3.3% and 0 ± 0% at 24 and 48 h respectively (Fig. 1).

### Cellular response to *Aspergillus* infection

**Fig. 2:**
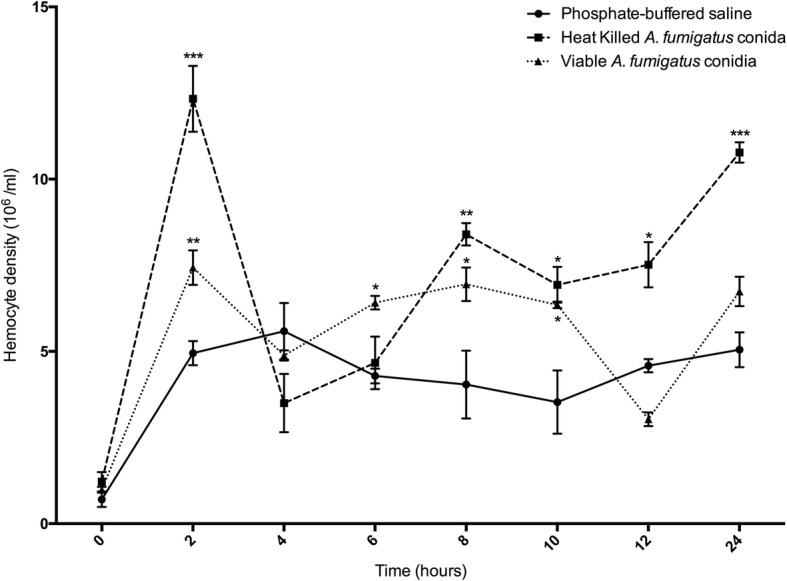
Alteration in circulating hemocyte density following inoculation with viable and heat killed *A. fumigatus* conidia. *G. mellonella* larvae were inoculated with 20 μl heat killed conidia *(*1 × 10^6^), viable *A. fumigatus* conidia *(*1 × 10^6^) or PBS and hemocytes were extracted and enumerated from 0 h to 24 h post inoculation. Statistical analysis was performed by comparing treatments to PBS injected controls at respective time points, (*: *p* < 0.05, **: *p* < 0.01, ***: *p* < 0.001). All values are the mean ± S.E of three independent experiments

Inoculation of larvae with viable conidia (1 × 10^6^/ larva) induced an initial spike in hemocyte density at 2 h (7.43 ± 0.50 × 10^6^, *p* < 0.01) which stabilized at 4 h (4.88 ± 0.15 × 10^6^). Hemocyte density increased at 6 (6.42 ± 0.20 × 10^6^, *p* < 0.05) and 8 h (6.95 ± 0.48 × 10^6^, *p* < 0.05). At 12 (3.03 ± 0.20 × 10^6^) and 24 h (6.74 ± 0.43 × 10^6^) post infection there were fluctuations in hemocyte density. Inoculation of larvae with heat killed conidia (1 × 10^6^/ larva) also produced similar cellular responses. Notably, hemocyte density peaked at 2 h (12.33 ± 0.95 × 10^6^, *p* < 0.001) but fell at 4 h (3.5 ± 0.85 × 10^6^) and did not decrease at 12 h (7.52 ± 0.66 × 10^6^) as observed with viable conidia (Fig. 2).

**Fig. 3:**
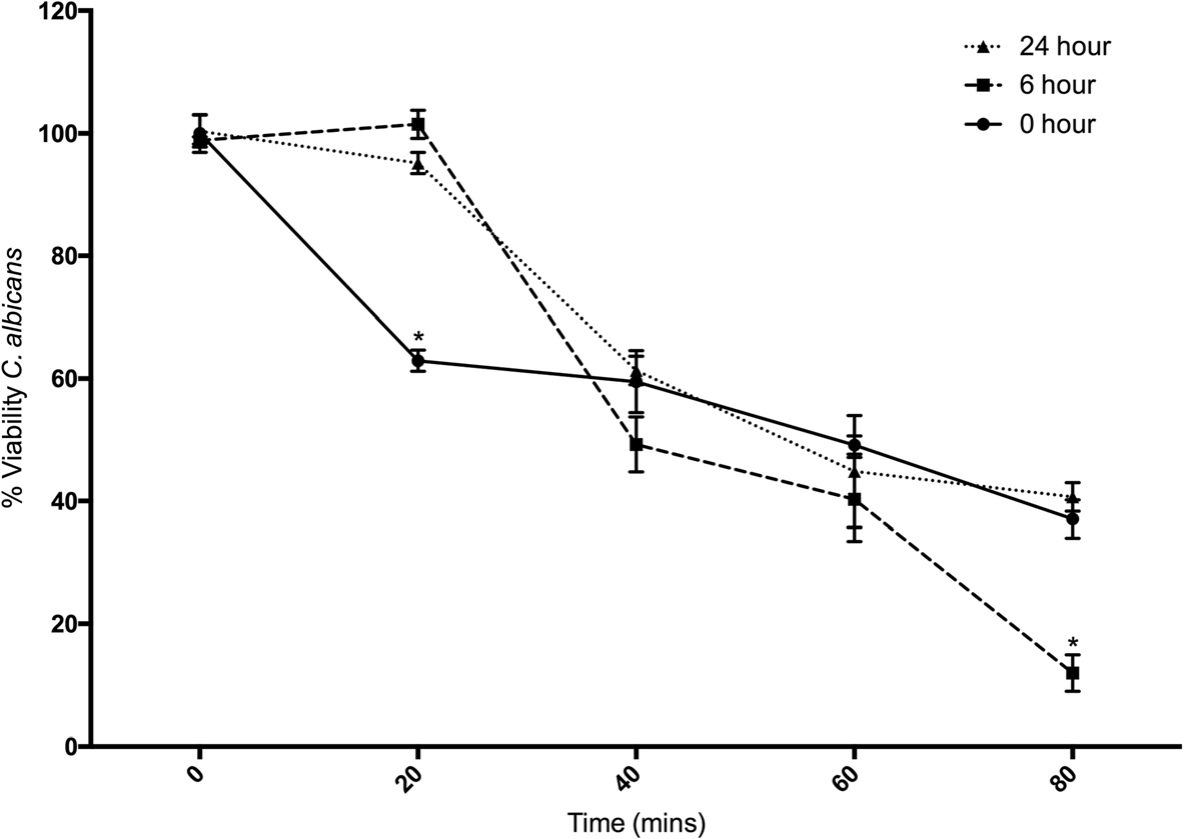
Fungicidal activity of hemocytes extracted from *G. mellonella* larvae at 0, 6 and 24 h after inoculation with *A. fumigatus* heat killed conidia (1 × 10^6^/ larvae). Opsonized *C. albicans* cells were incubated with hemocytes (2:1 ratio) for 80 min and aliquots taken every 20 min diluted and plated on YEPD agar plates. (*: *p* < 0.05). All values are the mean ± S.E of three independent experiments

Changes in hemocyte function following inoculation of larvae with heat killed *A. fumigatus* conidia were characterized by an ex vivo hemocyte microbicidal activity assay. Hemocytes from larvae inoculated with heat killed conidia for 6 h significantly decreased the viability of *C. albicans* to 11.94 ± 5.17% (*p* < 0.05) as compared to hemocytes from controls (37.07 ± 5.38%) and larvae inoculated for 24 h (40.67 ± 4.04%), at 80 mins. Interesting, hemocytes from 6 (101.49 ± 3.96%, *p* < 0.05) and 24 (95.17 ± 3.06%, *p* < 0.05) hour inoculated larvae were slower at killing yeast cells at *t* = 20, compared to hemocytes from control larvae (62.93 ± 2.98%) (Fig. 3).

### Modeling invasive and disseminated aspergillosis in *G. mellonella*

**Fig. 4:**
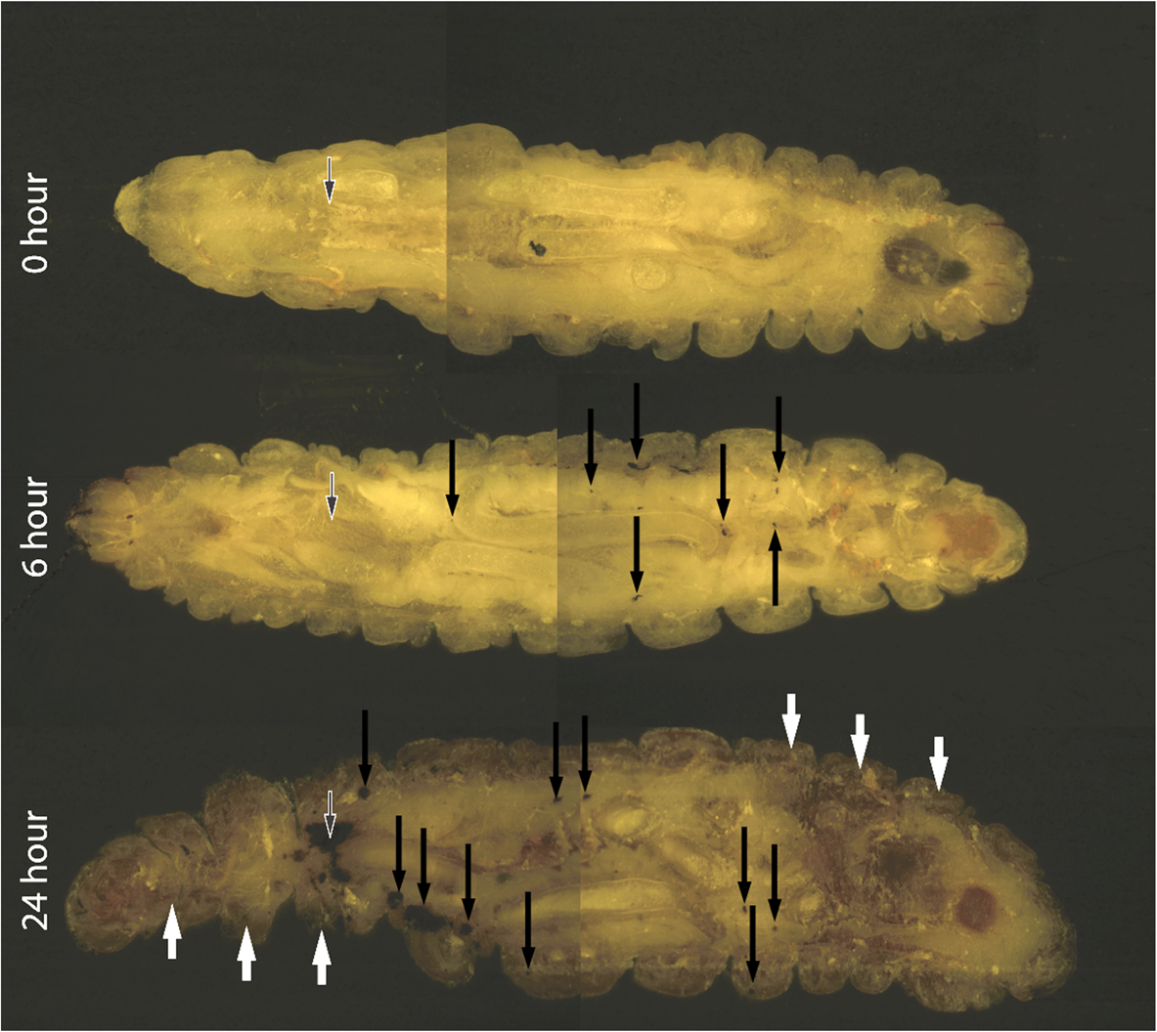
Cryoviz visualization of the stages of invasive and disseminated aspergillosis in *G. mellonella* after 6 and 24 h infection. Larvae were inoculated with 1 × 10^6^ viable *A. fumigatus* conidia for 6 and 24 h were embedded in Cryo-imaging embedding compound and sectioned (10 μm) using a Cryoviz™ (Bioinvision Inc., Cleveland, OH) cryo-imaging system. (Point of inoculation (white-edged arrow), fungal nodules/granulomas (black arrow), cuticle melanization (white arrows)

Cryo-imaging was employed to visualize the stages of invasive *A. fumigatus* disease in *G. mellonella* larvae. Larvae were inoculated with viable *A. fumigatus* conidia through the last left proleg in the posterior region (see white-edged arrow). Small discrete nodules appeared in the anterior region and around the perimeter of the haemocoel 6 h post infection (see black arrows) indicating dissemination of the *A. fumigatus* conidia from the site of infection. By 24 h there is extensive melanization of larval tissue and cuticle (see white arrows) indicating invasion from the insect haemocoel into surrounding tissue, and formation of both large diffuse fungal nodules at the site of inoculation and smaller distinct fungal nodules throughout the larva (Fig. 4).

**Fig. 5:**
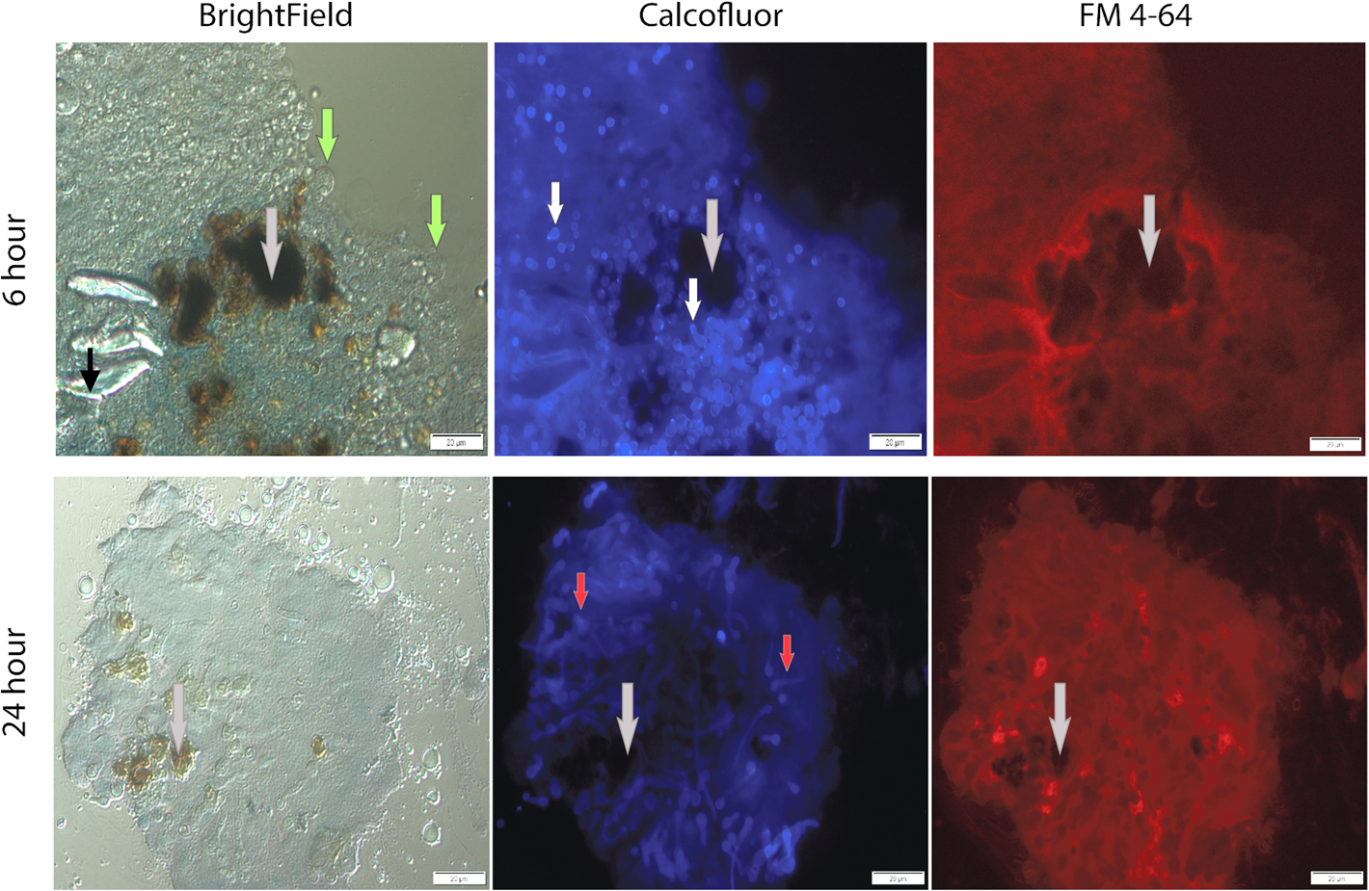
Visualization of development of *A. fumigatus* conidia and hyphae in fungal nodules/granulomas in *G. mellonella* larvae inoculated with 1 × 10^6^ viable conidia. Fungal nodules were dissected from larvae and stained with Calcofluor white and FM 4–64. Bright field images reveal the formation of melanized plaques (grey) and cellular infiltrates (green arrow). Fluorescent microscopy using Calcofluor white fluorescence revealed conidia germinating conidia (oval shaped; white arrow) at 6 h and dense hyphal infiltration (red arrow) at 24 h post infection. Corresponding images obtained from fungal nodules stained with FM 4–64 demonstrates the accumulation of hemocyte membrane at 6 and 24 h (scale bar corresponds to 20 μm)

Melanized lesions from larvae infected with *A. fumigatus* for 6 and 24 h were extracted and dissected. Dense hemocyte infiltration (green arrow) and melanized plaques (gray arrow) were visible by light microscope (Fig. 5). Confocal laser scanning microscopy of nodules isolated at 6 and 24 h and analysis of fluorescence due to calcofluor white binding confirmed the presence of germinated conidia (germ tube; black arrow) at 6 h and dense hyphal infiltration (white arrow) at 24 h post infection (Additional file 1: Figure S1). Interestingly, the use of fluorescence microscopy at 6 and 24 h revealed the presence of germinated conidia (white arrow) and hyphae (red arrow), respectively and fungal nodules stained with FM 4–64 illustrated the presence of hemocyte membrane (Fig. 5). Incubation of hemocytes with *A. fumigatus* (2:1 ratio) for 20 min suggest hemocyte phagocytosis (white arrow) and lysis around conidia (blue arrow) as well as attachment of viable hemocytes to the outer perimeter (red arrow) as evident from bright-field and FM 4–64 stained images (Additional file 2: Figure S2) and reported elsewhere [28]. Conidia and hyphae remained viable inside granulomatous tissue as determined via plate assay.

### Analysis of alterations in *G. mellonella* proteome following *A. fumigatus* infection

Label free quantitative proteomic analysis was performed on the *G. mellonella* cell free hemolymph after exposure of larvae to viable *A. fumigatus conidia* (1 × 10^6^/ larva) for 0, 6 and 24 h. In total, 2517 peptides were identified representing 252 proteins with two or more peptides and 28 and 108 (6 h v 0 h and 24 h v 0 h, respectively) proteins determined to be differentially abundant (ANOVA, *p* < 0.05) with a fold change of > 2. A total of 17 proteins were deemed exclusive (i.e. with LFQ intensities present in all four replicates of one treatment and absent in all four replicates of the other two treatments). These proteins were also used in statistical analysis of the total differentially expressed group following imputation of the zero values as described. After data imputation these proteins were included in subsequent statistical analysis. A principal component analysis (PCA) performed on all filtered proteins distinguished the 0, 6 and 24 h *A. fumigatus* treated samples indicating a clear difference between each proteome (Additional file 3: Figure S3A). Hierarchical clustering of z-score normalized intensity values for all significantly differentially abundant proteins (*n* = 252) produced the three replicates of each sample group (Additional file 3: Figure S3B). Furthermore, 2 major protein clusters were identified: 0 h and 6 h abundant proteins (Cluster A) and 24 h abundant proteins (Cluster B).

**Fig. 6:**
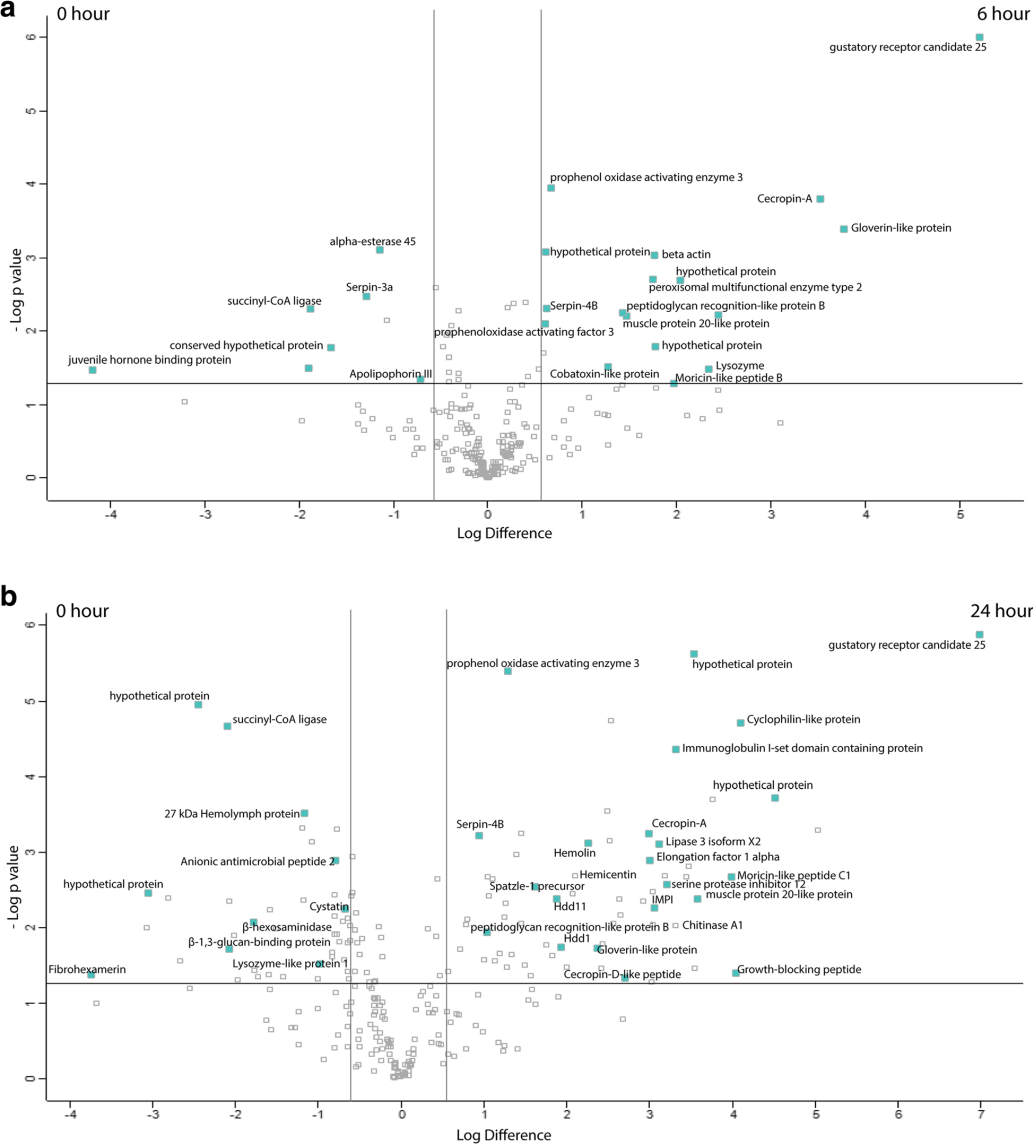
Proteomic responses of *G. mellonella* larvae following infection by 1 × 10^6^ viable *A. fumigatus* conidia after 6 (**a**) and 24 (**b**) hours. Volcano plots represent protein intensity difference (− log_2_ mean intensity difference) and significance in differences (− log *P*-value) based on a two-sided *t*-test. Proteins above the line are considered statistically significant (*p* value < 0.05) and those to the right and left of the vertical lines indicate relative fold changes > 2. Annotations are given for the most differentially abundant proteins identified in hemolymph from larvae infected with *A. fumigatus* for 6 and 24 h. These plots are based upon post imputed data

Proteins increased in relative abundance in larvae 6 h after infection with 1 × 10^6^ conidia versus non-infected larvae were gustatory receptor candidate 25 (37 fold), gloverin-like protein (14 fold), cecropin-A (11 fold), lysozyme (5 fold), moricin-like peptide B (4 fold), muscle protein 20-like protein (3 fold), peptidoglycan recognition-like protein B (3 fold) and prophenoloxidase activating enzyme 3 (2 fold). Proteins decreased in relative abundance in 6 h infected larvae compared to control larvae were juvenile hormone binding protein (18 fold), succinyl-CoA ligase (4 fold), serpin 3a (2 fold), alpha-esterase 45 (2 fold) and apolipophorin III (2 fold), (Fig. 6a).

Proteins increased in relative abundance in larvae exposed to *A. fumigatus* for 24 h versus control larvae were gustatory receptor candidate 25 (126 fold), moricin-like peptide D (33 fold), DNA-directed RNA polymerase II subunit RPB (22 fold), growth-blocking peptide (16 fold), moricin-like peptide C1 (16 fold), muscle protein 20-like protein (12 fold), protease inhibitor 1 precursor (12 fold), chemosensory protein (11 fold), Immunoglobulin I-set domain containing protein (10 fold), chitinase A1 (10 fold), Inducible metalloproteinase inhibitor protein (8 fold), cecropin-A (8 fold), cecropin-D-like peptide (7 fold), peptidoglycan-recognition protein-LB (6 fold), gloverin-like protein (5 fold), hemolin (5 fold), hemicentin (4 fold), spatzle-1 precursor (4 fold) and immune-related Hdd1 (4 fold). Proteins decreased in relative abundance in larvae 24 h post infection versus control larvae were fibrohexamerin (13 fold), dimeric dihydrodiol dehydrogenase (8 fold), glyceraldehyde-3-phosphate dehydrogenase (6 fold), β-1,3-glucan-binding protein (4 fold), C-type lectin 10 precursor (2 fold) and ecdysteroid-regulated 16 kDa protein (2 fold), (Fig. 6b).

## Discussion

*G. mellonella* larvae are widely utilized in determining in vivo antimicrobial drug activity, in assessing relative toxicity of compounds and in quantifying the virulence of bacterial and fungal pathogens [2, 29–31]. While *G. mellonella* larvae offer many advantages as in vivo models, there is the possibility of extending their use for modeling disease processes [2, 3]. This creates an opportunity to model infection processes in larvae that show similarities to those in mammals and to design therapies in the insect system that may be translated into mammals. The results presented here characterize the immune response of *G. mellonella* to *A. fumigatus* and describe the dissemination of the fungus through the larva in the first 24 h of infection. Inoculation of viable conidia (1 × 10^6^/ larva) resulted in a significant increase in the density of circulating hemocytes at 2 h as compared to the PBS control possibly due to the release of sessile hemocytes normally attached to the inner surface of the hemocoel or fat body of larvae [32]. Heat killed conidia produced a greater increase in hemocyte density and it is possible that loss of the rodlet/hydrophobin layer on their surface may trigger a larger immune response [33]. Heat killed conidia and germinating conidia display β-glucan on their cell surface and recruit neutrophils into the airways of C57BL/6 mice [34]. Hemocyte density was significantly elevated for 6, 8 and 10 h post infection with viable conidia and this may be associated with the formation of hyphae and secretion of metabolites from *A. fumigatus*. Hemocyte populations from larvae inoculated with heat killed *A. fumigatus* for 6 h displayed increased fungicidal activity as compared to hemocyte populations from control and 24 h treated larvae.

In mice, *A. fumigatus* increased the activity of both macrophages and neutrophils, which are essential in conidia and hyphal destruction by phagolysosome mediated killing, degranulation and NET formation [35, 36]. Interestingly, mice exposed to conidia show a significant increase in total immune cell density (in bronchoalveolar lavage fluid) but specifically an increase in macrophages after 4 h and neutrophils 48 h post exposure [37]. Histology of the CGD invasive aspergillosis mouse lung shows germinated conidia early and the formation of hyphae after 24 h with neutrophil infiltration and pyogranulomatous lesions surrounded by granulocytes [23].

Cryoviz imaging and fluorescent microscopy characterized the dissemination and development of *Aspergillus* within larvae. By 6 h, fungal infection had spread to distal sites of the larvae and is marked by the formation of well-defined melanized nodules consisting of granulocyte infiltration and encapsulation of *A. fumigatus* germinating conidia. Here, hemocytes accumulate around antigenic *A. fumigatus* conidia preventing its spread. Hemocyte membrane was found surrounding *A. fumigatus* conidia and hyphae which is consistent with encapsulation responses of *G. mellonella* larvae to foreign material [18]. Larvae at twenty four hours post infection were characterized by melanization of the cuticle, the appearance of de novo nodules and expansion of fungal nodules from the site of inoculation throughout the host. These nodules show similarities to granulomatous structures which are characteristic of invasive aspergillosis in the CGD murine model. Histology from the CGD murine lung illustrates granulomatous lesions with significant hyphal invasion [23]. However, insect granulomatous structures lack lymphocytes, key components of mammalian granulomas. *A. fumigatus* remained viable and grew through melanized nodules 6 and 24 h post infection thus highlighting the importance of nodulation as an early response to an invading fungal inoculum.

Quantitative proteomics was employed to characterize the larval humoral immune response to *A. fumigatus*. At 6 h there was an increase in the abundance of antimicrobial peptides and proteins (gloverin, moricin, lysozyme, cecropin) and proteins associated with the prophenoloxidase cascade (serpin-4B, prophenoloxidase activating enzyme 3 and prophenoloxidase activating factor 3) in hemolymph. During the mammalian innate response to *A. fumigatus,* a range of antimicrobial peptides (defensins, cathelicidins) and proteins (lactoferrin, lysozyme) are produced and these are essential in curtailing early fungal establishment and growth [38, 39]. Furthermore, the prophenoloxidase cascade is analogous to the mammalian complement protein cascade in terms of protein structure, function and mode of action [40, 41].

At 24 h post infection antimicrobial peptides and prophenoloxidase family members are increased but also proteins associated with tissue invasion (muscle protein 20 like protein), recognition and opsonization of fungal cells (hemolin, peptidoglycan recognition like protein B) and inhibition of fungal proteinases (insect metalloproteinase inhibitor (IMPI)) [42, 43]. IMPI is induced following fungal infection in *G. mellonella* and functions to inhibit the activity of secreted metalloproteinases which act as virulence factors to degrade host defenses [44]. *A. fumigatus* produces a variety of metalloproteinases most notably is Asp f5/mep, a 42 kDa Zn/Mep which possesses collagenlytic and elastinolytic activity and is important for immune cell recruitment in the murine lung [45, 46]. Gustatory protein was increased in abundance at 6 and 24 h post *A. fumigatus* infection and was also increased in *G. mellonella* larvae in response to entomopathogenic fungal culture filtrate and is hypothesized to be associated with altered feeding responses and possibly toxin avoidance in insects [47, 48]. Hdd1 and Hdd11 were found increased in 24 h *A. fumigatus* infected larval hemolymph. Both are induced following bacterial infection in *Hyphantria cunea* with the former sharing homology with mucin-5 AC-like protein from *Plutella xylostella* and the later homologous with Noduler from *Antheraea mylitta* [49, 50]*.* Noduler shares a reeler domain with Hdd11 and binds both insect hemocytes and fungal β-1, 3 glucan, is enriched in nodules and may act as a facilitator of nodulation [51]. Interestingly, 27 kDa hemolymph protein, Lysozyme–like protein 1, C-type lectin 10 precursor and β-glucan binding protein were significantly decreased at 24 h possibly as a result of binding to hyphal structures and facilitating hemocyte recognition of fungal structures. Anionic antimicrobial peptide 2 was decreased in abundance 24 h after *A. fumigatus* infection (Fig. 6b) and was also decreased in *G. mellonellla* infected with *C. albicans* [52]. At 6 h post infection there is a decrease in Apolipophorin III in hemolymph. Apolipophorin III mediates recognition of fungal conidia, activates the phenoloxidase cascade and dose dependently enhances melanized nodule formation [53, 54]. Its decreased abundance in hemolymph may be as a result of increased binding to *A. fumigatus* at the site of nodule formation [55].

## Conclusion

Due to the lack of a comparable respiratory system to mammals, the use of *G. mellonella* to study *A. fumigatus* interactions at the bronco – alveolar surface is not possible. However, the results presented here, reveal similarities between the development of invasive aspergillosis in mammals and in *G. mellonella* larvae. This work has characterized the cellular and humoral immune responses of *G. mellonella* larvae to *A. fumigatus* as well as the morphological changes of conidia and dissemination of hyphae throughout larvae. These results document significant parallels between the mammalian and insect responses to *A. fumigatus* infection, and illustrate how the development of invasive aspergillosis in larvae shows similarities to that which occurs in mammals. Studying the development of invasive aspergillosis in larvae may give novel insights into the pathogen – host interactions that could improve our understanding of this disease process in humans.

## Methods

### *A. fumigatus* culture conditions

*A. fumigatus* ATCC 26933 (obtained from the American Type Culture Collection) was used in this study. *Aspergillus* cultures were grown in sabouraud dextrose broth (SAB) (Sigma Aldrich) at 37 °C and 200 rpm for up to 2 days. Stocks were maintained on malt extract agar (MEA) (Oxoid).

### Larval culture and inoculation

Sixth instar larvae of the greater wax-moth *G. mellonella* (Livefoods Direct Ltd., Sheffield, England), were stored in the dark at 15 °C to prevent pupation. Larvae weighing 0.22 ± 0.03 g were selected and used within 2 weeks of receipt. Ten healthy larvae per treatment and controls (20 μl PBS for appropriate incubation time); (*n* = 3) were placed in sterile 9 cm Petri dishes lined with Whatman filter paper and containing wood shavings. Larvae were inoculated with viable or non-viable (heat-killed) conidia through the last left pro-leg into the hemocoel with a Myjector U-100 insulin syringe (Terumo Europe N.V., Belgium). Larvae were acclimatized to 37 °C for 1 h prior to all experiments and incubated at 37 °C for all studies. All experiments were performed independently on three separate occasions.

### Determination of hemocyte density

Larvae were inoculated with 20 μl of phosphate-buffered saline (PBS) containing heat killed, PBS containing viable *A. fumigatus* (1 × 10^6^) conidia or PBS. Heat killed (HK) conidia were produced by heating to 95 °C for 20 min and plated on SAB agar to confirm loss of viability. Changes in hemocyte density were assessed by bleeding 40 μl each from 5 *G. mellonella* larvae into a micro-centrifuge tube on ice, to prevent melanization. Hemolymph was diluted in 0.37% (*v*/v) mercaptoethanol supplemented PBS and cell density was determined using a hemocytometer. Cell density was expressed in terms of hemocytes per ml of hemolymph. Statistical analysis was performed by comparing treatments to PBS injected controls at respective time points. Experiments were performed on three independent occasions and the means ± S.E. were determined.

### Determination of fungicidal activity of hemocytes

Larvae were inoculated with 1 × 10^6^ heat killed conidia and incubated for 0, 6 or 24 h (*n* = 10) at 37 °C. Hemocytes were extracted, washed and mixed with cell free hemolymph opsonized *C. albicans* (2 × 10^6^ cells) at 37 °C at a 2:1 ratio (hemocytes: yeast cells) in PBS as previously described [8]. Aliquots were taken at 0, 20, 40, 60 and 80 min, diluted and plated onto YEPD agar plates. Colonies were counted and expressed in terms of percentage of the original number of cells at time zero. Results were calculated as the mean (± S.E.) from at least three experiments with colony counts performed in triplicate for each sample.

### Cryo-imaging of *A. fumigatus* infection in *G. mellonella*

*G. mellonella* were inoculated with 1 × 10^6^ viable *A. fumigatus* conidia for 0, 6 and 24 h. Larvae were placed on ice to inhibit any movement. They were embedded in Bioinvision Cryo-Imaging Embedding Compound and flash-frozen in liquid nitrogen and later mounted on a stage for sectioning. Sectioning and imaging was carried out every 10 μm using a Cryoviz™ (Bioinvision Inc., Cleveland, OH) cryo-imaging system.

### Fluorescent imaging of fungal nodules

*G. mellonella* larvae infected with *A. fumigatus* for 6 and 24 h were dissected in PBS and nodules dissected apart with fine needles, transferred to a glass slide, washed with PBS 3 times and stained with Calcofluor white and FM 4–64 (Sigma) for 30 min at 16 °C. The cells were washed twice (PBS) and a cover slide was placed on top. The cover slides were then fixed in-situ by applying a clear sealing solution around the perimeter of the slide which also prevented the sample drying out. Cells were viewed with an Olympus Fluoview 1000 Confocal microscope and an Olympus BX61 fluorescent microscope.

### Label free quantitative proteomics of larval hemolymph

Shotgun quantitative proteomics was conducted on hemocyte-free hemolymph from larvae (*n* = 10) at 0, 6 and 24 h post infection with viable *A. fumigatus* (1 × 10^6^/ larva). Protein (75 μg) was prepared, identified and analyzed according to established protocols and procedures [3].

Briefly, protein identification from the MS/MS data was performed using the Andromeda search engine in MaxQuant (version 1.2.2.5) and the data correlated against a 6-frame translation of the EST contigs for *G. mellonella* [56, 57]*.* The MS proteomic data and MaxQuant search output files have been added to the ProteomeXchange Consortium [58] via the PRIDE partner repository with the dataset identifier PXD008196.

The Perseus software package (v. 1.5.5.3) was used for results processing, statistical analyses and graphics generation. LFQ intensities were log_2_-transformed and ANOVA of significance and t-tests between the hemolymph proteomes of 0, 6 and 24 h *A. fumigatus* treated larvae was performed using a *p*-value of 0.05 and significance was determined using FDR correction (Benjamini-Hochberg). Proteins which had non-existent values (suggestive of absence or very low abundance in a sample) were also used in statistical analysis Proteins found to be absent (below the level of detection) in one or more treatments and present (above the level of detection) in three or fewer treatments were also used in statistical analysis of the total differentially expressed group following imputation of the zero values with values that simulate low abundant proteins.

### Statistical analysis

All experiments were performed on three independent occasions and results are expressed as the mean ± S.E. Analysis of changes in hemocyte density and protein abundance were performed by One-way ANOVA. All statistical analysis listed performed using GraphPad Prism. Differences were considered significant at *p* < 0.05.

## Additional files

Additional file 1: **Figure S1.** Visualization of development of *A. fumigatus* conidia and hyphae in fungal nodules/granulomas in *G. mellonella* larvae inoculated with 1 × 10^6^ viable conidia. Fungal nodules were dissected from larvae and stained with Calcofluor white. Confocal laser scanning microscopy using Calcofluor white fluorescence revealed germinated conidia (germ tube) and germinating conidia (oval shaped) at 6 h and dense hyphal infiltration at 24 h post infection within nodules/granulomas (Black arrow; germinated conidia, white arrows; hyphae), (Scale bar corresponds to 10 μm). (TIF 1660 kb)

Additional file 2: **Figure S2.** The acute ex vivo cellular response of *G. mellonella* hemocytes to *A. fumigatus.* Hemocytes were extracted from *G. mellonella* washed 3 times with PBS and mixed for 20 min at a 2: 1 ratio with live *A. fumigatus* conidia. Bright field images suggest the phagocytosis (white arrow) and accumulation and lysis (Blue arrow) of hemocytes around conidia as well as viable hemocytes attached to the outer perimeter (red arrow), (Scale bar corresponds to 20 μm). (TIF 4357 kb)

Additional file 3: **Figure S3.** Principal component analysis (PCA) and hierarchical clustering of *G. mellonella* hemolymph proteomic profiles following infection with viable *A. fumigatus* conidia for 0, 6 and 24 h. (A) PCA of four replicates of each treatment included in LFQ analysis with a clear distinction between each time point. (B) Two-way unsupervised hierarchical clustering of the median protein expression values of all statistically significant differentially abundant proteins. Hierarchical clustering (columns) identified 2 distinct clusters comprising the four replicates from their original sample groups. (TIF 4138 kb)

## Abbreviations

AMP: Antimicrobial peptide
CGD: Chronic granulomatous disease
DEP: Differentially expressed proteins;
FDR: False Discovery Rates
LFQ: Label-free quantification
MPO: Myeloperoxidase
SSDA: Statistically significant differentially abundant

## References

[1] Cotter G, Doyle S, Kavanagh K (2000). Development of an insect model for the in vivo pathogenicity testing of yeasts. FEMS Immunol Med Microbiol.

[2] Mukherjee K, Hain T, Fischer R, Chakraborty T, Vilcinskas A (2013). Brain infection and activation of neuronal repair mechanisms by the human pathogen listeria monocytogenes in the lepidopteran model host galleria mellonella. Virulence.

[3] Sheehan G, Kavanagh K (2018). Analysis of the early cellular and humoral responses of galleria mellonella larvae to infection by Candida albicans. Virulence.

[4] Mylonakis E (2008). Galleria mellonella and the study of fungal pathogenesis: making the case for another genetically tractable model host. Mycopathologia.

[5] Kavanagh K, Reeves EP (2004). Exploiting the potential of insects for in vivo pathogenicity testing of microbial pathogens. FEMS Microbiol Rev.

[6] Fuchs BB, O’Brien E, Khoury JB, Mylonakis E (2010). Methods for using galleria mellonella as a model host to study fungal pathogenesis. Virulence.

[7] Bergin D, Brennan M, Kavanagh K (2003). Fluctuations in haemocyte density and microbial load may be used as indicators of fungal pathogenicity in larvae of galleria mellonella. Microbes Infect.

[8] Bergin D, Reeves EP, Renwick J, Frans B, Kavanagh K, Wientjes FB (2005). Superoxide production in galleria mellonella Hemocytes : identification of proteins homologous to the NADPH oxidase complex of human neutrophils Superoxide Production in Galleria mellonella Hemocytes : Identification of Proteins Homologous to the NADPH Ox. Infect Immun.

[9] Slater JL, Gregson L, Denning DW, Warn PA (2011). Pathogenicity of *Aspergillus fumigatus* mutants assessed in *Galleria mellonella* matches that in mice. Med Mycol.

[10] Brennan M, Thomas DY, Whiteway M, Kavanagh K (2002). Correlation between virulence of Candida albicans mutants in mice and galleria mellonella larvae. FEMS Immunol Med Microbiol.

[11] Mylonakis E, Moreno R, El Khoury JB, Idnurm A, Heitman J, Calderwood SB (2005). Galleria mellonella as a model system to study Cryptococcus neoformans pathogenesis. Infect Immun.

[12] Browne N, Heelan M, Kavanagh K (2013). An analysis of the structural and functional similarities of insect hemocytes and mammalian phagocytes. Virulence.

[13] Wojda I (2017). Immunity of the greater wax moth galleria mellonella. Insect Sci.

[14] Tojo S, Naganuma F, Arakawa K, Yokoo S (2000). Involvement of both granular cells and plasmatocytes in phagocytic reactions in the greater wax moth, galleria mellonella. J Insect Physiol.

[15] Fuchs BB, Mylonakis E (2006). Using non-mammalian hosts to study fungal virulence and host defense. Curr Opin Microbiol.

[16] Brinkmann V, Reichard U, Goosmann C, Fauler B, Uhlemann Y, Weiss DS (2004). Neutrophil extracellular traps kill Bacteria. Science.

[17] Altincicek B, Stotzel S, Wygrecka M, Preissner KT, Vilcinskas A (2008). Host-derived extracellular nucleic acids enhance innate immune responses, induce coagulation, and prolong survival upon infection in insects. J Immunol.

[18] Dubovskiy I, Kryukova N, Glupov V, Ratcliffe N (2016). Encapsulation and nodulation in insects. ISJ.

[19] Fallon JP, Reeves EP, Kavanagh K (2010). Inhibition of neutrophil function following exposure to the aspergillus fumigatus toxin fumagillin. J Med Microbiol.

[20] Renwick J, Reeves EP, Wientjes FB, Kavanagh K (2007). Translocation of proteins homologous to human neutrophil p47phox and p67phox to the cell membrane in activated hemocytes of galleria mellonella. Dev Comp Immunol.

[21] Dagenais TRT, Keller NP (2009). Pathogenesis of aspergillus fumigatus in invasive aspergillosis. Clin Microbiol Rev.

[22] Margalit A, Kavanagh K (2015). The innate immune response to aspergillus fumigatus at the alveolar surface. FEMS Microbiol Rev.

[23] Dennis CG, Greco WR, Brun Y, Youn R, Slocum HK, Bernacki RJ (2006). Effect of amphotericin B and micafungin combination on survival, histopathology, and fungal burden in experimental aspergillosis in the p47 phox−/− mouse model of chronic granulomatous disease. Antimicrob Agents Chemother.

[24] O’Hanlon KA, Cairns T, Stack D, Schrettl M, Bignell EM, Kavanagh K (2011). Targeted disruption of nonribosomal peptide synthetase Pes3 augments the virulence of aspergillus fumigatus. Infect Immun.

[25] Renwick J, Daly P, Reeves EP, Kavanagh K (2006). Susceptibility of larvae of galleria mellonella to infection by aspergillus fumigatus is dependent upon stage of conidial germination. Mycopathologia.

[26] van de Veerdonk FL, Gresnigt MS, Romani L, Netea MG, Latgé J-P (2017). Aspergillus fumigatus morphology and dynamic host interactions. Nat Rev Microbiol.

[27] Maurer E, Browne N, Surlis C, Jukic E, Moser P, Kavanagh K (2015). Galleria mellonella as a host model to study aspergillus terreus virulence and amphotericin B resistance. Virulence.

[28] Schmit AR, Ratcliffe NA. The encapsulation of foreign tissue implants in galleria mellonella larvae. J Insect Physiol. 1977;23 10.1016/0022-1910(77)90027-0 323370

[29] Tsai CJ-Y, Lo JMS, Proft T (2016). *Galleria mellonella* infection models for the study of bacterial diseases and for antimicrobial drug testing. Virulence.

[30] Hornsey M, Wareham DW (2011). In vivo efficacy of glycopeptide-colistin combination therapies in a galleria mellonella model of Acinetobacter baumannii infection. Antimicrob Agents Chemother.

[31] Desbois AP, Coote PJ (2012). Utility of greater wax moth larva (galleria mellonella) for evaluating the toxicity and efficacy of new antimicrobial agents. Adv Appl Microbiol.

[32] Ratcliffe NA (1985). Invertebrate immunity - a primer for the non-specialist. Immunol Lett.

[33] Aimanianda V, Bayry J, Bozza S, Kniemeyer O, Perruccio K, Elluru SR (2009). Surface hydrophobin prevents immune recognition of airborne fungal spores. Nature.

[34] Hohl TM, Van Epps HL, Rivera A, Morgan LA, Chen PL, Feldmesser M (2005). Aspergillus fumigatus triggers inflammatory responses by stage-specific β-glucan display. PLoS Pathog.

[35] Philippe B, Ibrahim-Granet O, Prévost MC, Gougerot-Pocidalo MA, Perez MS, Van der Meeren A (2003). Killing of aspergillus fumigatus by alveolar macrophages is mediated by reactive oxidant intermediates. Infect Immun.

[36] Bellocchio S, Moretti S, Perruccio K, Fallarino F, Bozza S, Montagnoli C (2004). TLRs govern neutrophil activity in aspergillosis. J Immunol.

[37] Buskirk AD, Green BJ, Lemons AR, Nayak AP, Goldsmith WT, Kashon ML (2014). A murine inhalation model to characterize pulmonary exposure to dry aspergillus fumigatus conidia. PLoS One.

[38] Zhang Y, Wu J, Xin Z, Wu X (2014). Aspergillus fumigatus triggers innate immune response via NOD1 signaling in human corneal epithelial cells. Exp Eye Res.

[39] Alekseeva L, Huet D, Féménia F, Mouyna I, Abdelouahab M, Cagna A (2009). Inducible expression of beta defensins by human respiratory epithelial cells exposed to aspergillus fumigatus organisms. BMC Microbiol.

[40] Clow LA, Raftos DA, Gross PS, Smith LC (2004). The sea urchin complement homologue, SpC3, functions as an opsonin. J Exp Biol.

[41] Söderhäll K, Cerenius L (1998). Role of the prophenoloxidase-activating system in invertebrate immunity. Curr Opin Immunol.

[42] Gillespie and JP, Kanost MR, Trenczek T (1997). Biological mediators of insect immunity. Annu Rev Entomol.

[43] Gillespie JP, Bailey AM, Cobb B, Vilcinskas A (2000). Fungi as elicitors of insect immune responses. Arch Insect Biochem Physiol.

[44] Vertyporokh L, Wojda I (2017). Expression of the insect metalloproteinase inhibitor IMPI in the fat body of galleria mellonella exposed to infection with Beauveria bassiana. Acta Biochim Pol.

[45] Vasco P, Herriko E, Rementeria A, López-molina N, Ludwig A (2005). Genes and molecules involved in aspergillus fumigatus virulence genes and molecules involved in aspergillus fumigatus virulence. Rev Iberoam Micol.

[46] Namvar S, Warn P, Farnell E, Bromley M, Fraczek M, Bowyer P (2015). Aspergillus fumigatus proteases, asp f 5 and asp f 13, are essential for airway inflammation and remodelling in a murine inhalation model. Clin Exp Allergy.

[47] Chapman RF (2003). Contact chemoreception in feeding by phytophagous insects. Annu Rev Entomol.

[48] Mc Namara L, Carolan JC, Griffin CT, Fitzpatrick D, Kavanagh K (2017). The effect of entomopathogenic fungal culture filtrate on the immune response of the greater wax moth, galleria mellonella. J Insect Physiol.

[49] Shin SW, Park SS, Park DS, Kim MG, Kim SC, Brey PT (1998). Isolation and characterization of immune-related genes from the fall webworm, Hyphantria cunea, using PCR-based differential display and subtractive cloning. Insect Biochem Mol Biol.

[50] Sarauer BL, Gillott C, Hegedus D (2003). Characterization of an intestinal mucin from the peritrophic matrix of the diamondback moth, Plutella xylostella. Insect Mol Biol.

[51] Gandhe AS, John SH, Nagaraju J (2007). Noduler, a novel immune up-regulated protein mediates nodulation response in insects. J Immunol.

[52] Mak P, Zdybicka-Barabas A, Cytryńska M (2010). A different repertoire of galleria mellonella antimicrobial peptides in larvae challenged with bacteria and fungi. Dev Comp Immunol.

[53] Halwani AE, Niven DF, Dunphy GB (2000). Apolipophorin-III and the interactions of lipoteichoic acids with the immediate immune responses of galleria mellonella. J Invertebr Pathol.

[54] Whitten MMA, Tew IF, Lee BL, Ratcliffe NA (2004). A novel role for an insect apolipoprotein (Apolipophorin III) in −1,3-glucan pattern recognition and cellular encapsulation reactions. J Immunol.

[55] Fallon JP, Troy N, Kavanagh K (2011). Pre-exposure of galleria mellonella larvae to different doses of aspergillus fumigatus conidia causes differential activation of cellular and humoral immune responses. Virulence.

[56] Cox J, Neuhauser N, Michalski A, Scheltema RA, Olsen JV, Mann M (2011). Andromeda: a peptide search engine integrated into the MaxQuant environment. J Proteome Res.

[57] Vogel H, Altincicek B, Glöckner G, Vilcinskas A (2011). A comprehensive transcriptome and immune- gene repertoire of the lepidopteran model host galleria mellonella. BMC Genomics.

[58] Côté RG, Griss J, Dianes JA, Wang R, Wright JC, van den Toorn HWP (2012). The PRoteomics IDEntification (PRIDE) converter 2 framework: an improved suite of tools to facilitate data submission to the PRIDE database and the ProteomeXchange consortium. Mol Cell Proteomics.

