# Supplementary figures and images for: Characterisation of the cellular and proteomic response of Galleria mellonella larvae to the development of invasive aspergillosis
Source: BMC Microbiol. 2018 Jun 28;18:63. doi: 10.1186/s12866-018-1208-6 (PMC6025711; doi:10.1186/s12866-018-1208-6)

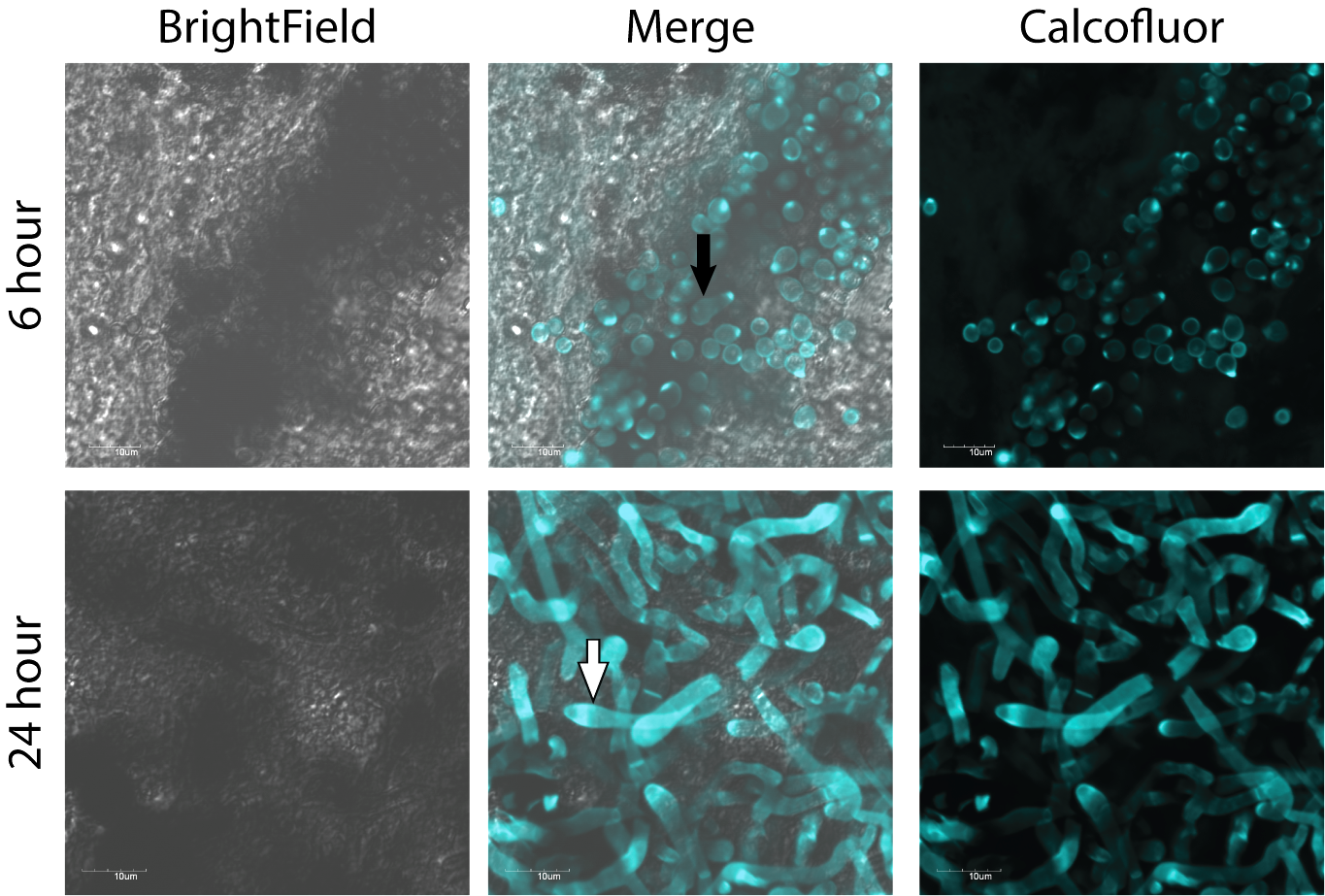

Supplement: Supplementary file 1 — Figure S1. Visualization of development of A. fumigatus conidia and hyphae in fungal nodules/granulomas in G. mellonella larvae inoculated with 1 × 10^6^ viable conidia. Fungal nodules were dissected from larvae and stained with Calcofluor white. Confocal laser scanning microscopy using Calcofluor white fluorescence revealed germinated conidia (germ tube) and germinating conidia (oval shaped) at 6 h and dense hyphal infiltration at 24 h post infection within nodules/granulomas (Black arrow; germinated conidia, white arrows; hyphae), (Scale bar corresponds to 10 μm). (TIF 1660 kb) [file 12866_2018_1208_MOESM1_ESM.tif]

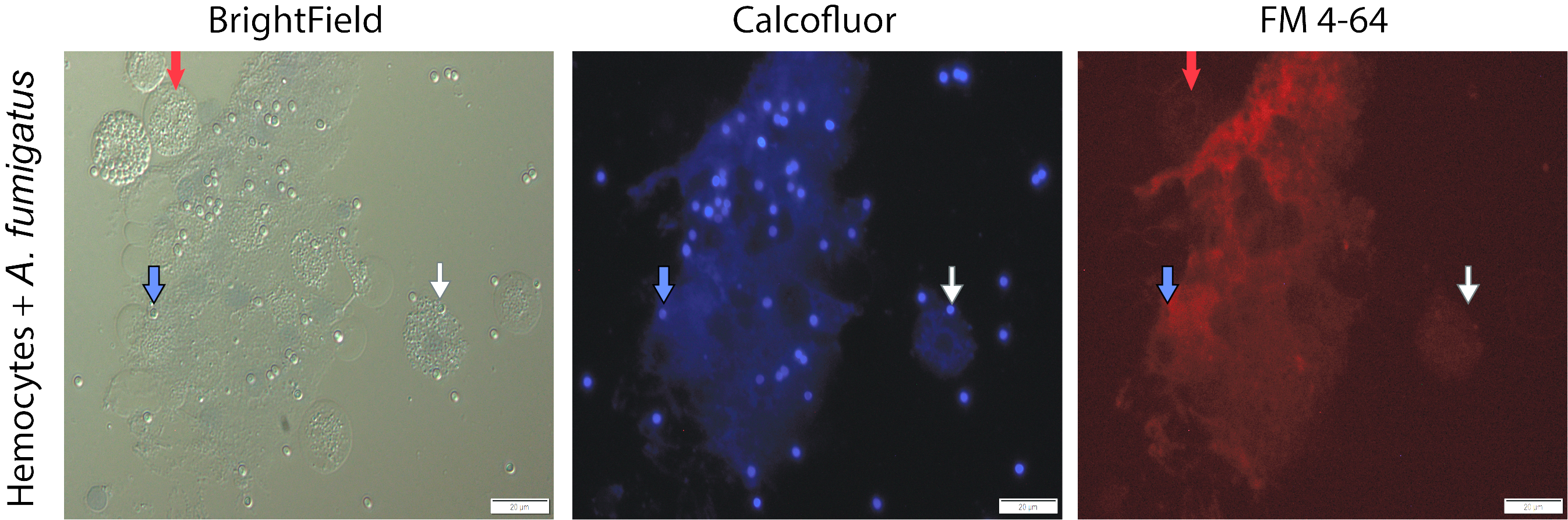

Supplement: Supplementary file 2 — Figure S2. The acute ex vivo cellular response of G. mellonella hemocytes to A. fumigatus. Hemocytes were extracted from G. mellonella washed 3 times with PBS and mixed for 20 min at a 2: 1 ratio with live A. fumigatus conidia. Bright field images suggest the phagocytosis (white arrow) and accumulation and lysis (Blue arrow) of hemocytes around conidia as well as viable hemocytes attached to the outer perimeter (red arrow), (Scale bar corresponds to 20 μm). (TIF 4357 kb) [file 12866_2018_1208_MOESM2_ESM.tif]

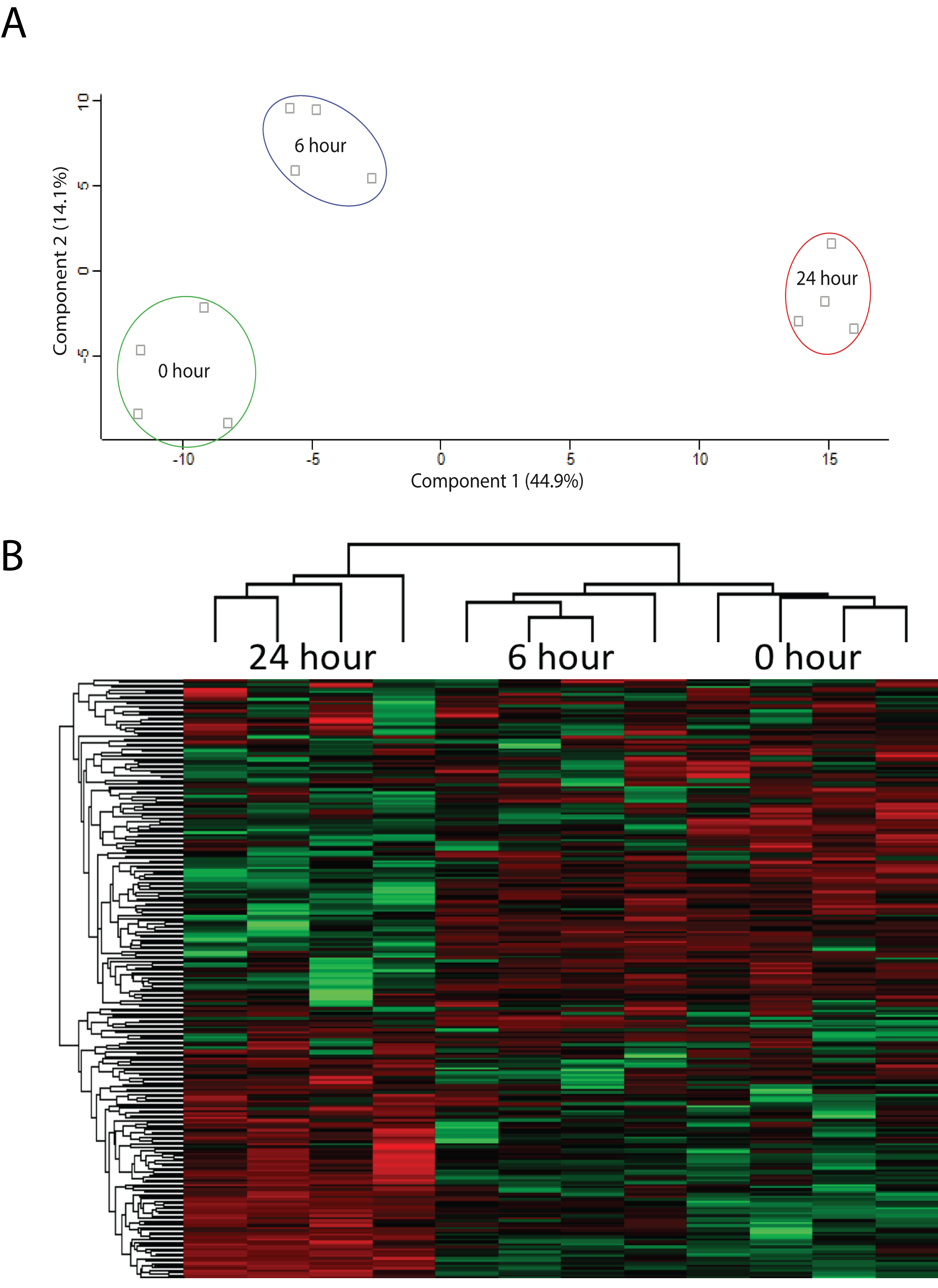

Supplement: Supplementary file 3 — Figure S3. Principal component analysis (PCA) and hierarchical clustering of G. mellonella hemolymph proteomic profiles following infection with viable A. fumigatus conidia for 0, 6 and 24 h. (A) PCA of four replicates of each treatment included in LFQ analysis with a clear distinction between each time point. (B) Two-way unsupervised hierarchical clustering of the median protein expression values of all statistically significant differentially abundant proteins. Hierarchical clustering (columns) identified 2 distinct clusters comprising the four replicates from their original sample groups. (TIF 4138 kb) [file 12866_2018_1208_MOESM3_ESM.tif]
